# Bronchoscopy and molecular diagnostic techniques to identify superimposed infections in COVID-19-associated ARDS: a case series from Ecuador during the second wave

**DOI:** 10.3389/fmed.2024.1409323

**Published:** 2024-10-30

**Authors:** Killen Harold Briones Claudett, Mónica H. Briones-Claudett, Roger Murillo Vasconez, Jaime G. Benitez Sólis, Killen H. Briones Zamora, Amado X. Freire, Pedro Barberan-Torres, Michelle Grunauer

**Affiliations:** ^1^School of Medicine, Universidad Internacional del Ecuador (UIDE), Quito, Ecuador; ^2^Intensive Care Unit, Ecuadorian Institute of Social Security (IESS), Babahoyo, Ecuador; ^3^Intensive Care Unit, Ecuadorian Institute of Social Security (IESS), Ecuadorian Social Security Institute (IESS), Babahoyo, Ecuador; ^4^Omni Hospital, Guayaquil, Ecuador; ^5^Facultad de Ciencias Médicas, Universidad de Especialidades Espíritu Santo, Guayaquil, Ecuador; ^6^Department of Medicine, University of Tennessee Health Science Center (UTHSC), Memphis, TN, United States; ^7^Universidad Internacional del Ecuador (UIDE), Quito, Ecuador; ^8^School of Medicine, Universidad San Francisco de Quito, Quito, Ecuador

**Keywords:** COVID-19, ARDS, bronchoscopy, molecular techniques (RFLP), superimposed infections, resource-limited settings (RLS), second wave

## Abstract

**Introduction:**

COVID-19-associated acute respiratory distress syndrome (CARDS) poses significant challenges in resource-limited settings. This case series explores the role of bronchoscopy and molecular techniques in identifying superimposed infections in CARDS patients during the second wave of the pandemic in Ecuador.

**Methods:**

Nine critically ill CARDS patients underwent bronchoscopy and molecular testing to detect co-infections and superinfections. Clinical presentations, diagnostic findings, and outcomes were analyzed.

**Results:**

Bronchoscopy and molecular techniques identified a range of secondary infections, including multidrug-resistant pathogens such as *Acinetobacter baumannii* and *Klebsiella pneumoniae*. The case series highlights the complexities of managing severe COVID-19 cases in resource-constrained environments.

**Discussion:**

Early identification of microorganisms using PCR methods allows for rapid and accurate diagnosis, facilitating targeted management of critically ill CARDS patients. The study underscores the importance of advanced diagnostic tools and adaptable strategies in pandemic situations, particularly in low-resource settings.

## Background

As of 2021, the global pandemic caused by severe acute respiratory syndrome coronavirus 2 (SARS-CoV-2), which first emerged in late 2019 in Wuhan, China, has resulted in at least 5 million deaths worldwide (1). The specific characteristics of severe COVID-19 led to the development of its nomenclature, CARDS, which refers to COVID-19-associated acute respiratory distress syndrome (2).

The clinical manifestations of SARS-CoV-2’s biochemical behavior in CARDS are characterized by low blood oxygen levels and systemic hypoxemia, justifying the use of supplemental oxygen across a wide range (3). To diagnose CARDS, the Berlin 2012 ARDS diagnostic criteria must be met (4). The use of serum biomarkers, such as IL-6, C-reactive protein, ferritin, d-dimer, white blood cell count, and lymphocyte count, is crucial for staging the infection (5).

The management of COVID-19-associated acute respiratory distress syndrome (CARDS) has been the subject of extensive research, particularly regarding the use of immunomodulatory agents. A recent open-label, randomized controlled trial compared the efficacy of tocilizumab and baricitinib in hospitalized patients with severe COVID-19 (6). The study, which included 251 patients with a PaO2/fraction of inspired oxygen (FiO2) ratio of <200, found that baricitinib was non-inferior to tocilizumab for the primary composite outcome of mechanical ventilation or death at day 28. These findings highlight the ongoing efforts to optimize treatment strategies for severe COVID-19.

SARS-CoV-2 is primarily transmitted from person to person through respiratory droplets larger than 5 μm, especially when the patient presents with respiratory symptoms such as coughing and sneezing, and through contact with contaminated surfaces or fomites. Consequently, when managing patients under investigation, probable, or confirmed cases, it is crucial to implement standard precautions, contact precautions, and droplet transmission precautions. This is particularly crucial for healthcare workers performing bronchoscopy who are at high risk of exposure and require adequate personal protective equipment (7).

While airborne transmission by droplet nuclei or aerosols (capable of transmitting over distances >2 m) has not been definitively demonstrated for SARS-CoV-2, it is believed that this mode of transmission could occur during invasive respiratory tract procedures such as bronchoscopy (8).

In addition, the timing of presentation of other respiratory pathogen infections alongside SARS-CoV-2 poses a challenge, which can be categorized as follows: 1) coinfection, which refers to the presence of another pathogen at the time of diagnosis of SARS-CoV-2 infection, and 2) superinfection, which refers to the occurrence of another pathogen during the course of SARS-CoV-2 infection (9). Survival rate, length of hospital stay, and sequelae are critical factors, regardless of whether a patient exhibits coinfection or superinfection. Furthermore, bronchoscopy and bronchoalveolar lavage (BAL) are powerful tools for sampling microbiological and molecular diagnostic testing, thereby identifying specific microorganisms growing in the bronchial environment and enabling targeted therapeutic interventions at the appropriate time (10).

During the second wave of COVID-19 in Ecuador, which lasted from November 2020 to May 2021, there was a significant increase in cases and hospitalizations. The peak occurred in mid-January 2021, with over 3,000 confirmed cases per week and nearly 1,600 hospitalizations (11). This context is crucial for understanding the cases presented in this study.

The role of bronchoscopy in the management of COVID-19 remains a subject of ongoing debate. For patients with severe COVID-19, particularly those admitted to the intensive care unit (ICU) (12), bronchoscopy may be essential to manage complications, such as atelectasis or hemoptysis, to address issues related to mechanical ventilation, and to identify superinfections. However, the use of bronchoscopy in COVID-19 patients is not without risk, notably the potential for viral transmission to healthcare staff. While various scientific societies have published guidelines to minimize heterogeneity in clinical practice (13), the evidence supporting these guidelines is limited and primarily consists of short series.

It is important to note that bronchoscopy can sometimes reveal unexpected findings in patients with suspected COVID-19 (14). In some cases, what initially appears to be an infection may be a different condition, such as vasculitis. A case report of granulomatosis with polyangiitis (GPA) presenting with a false positive COVID-19 antibody test highlights this potential for misdiagnosis (15). This underscores the importance of maintaining a broad differential diagnosis and the potential value of bronchoscopy in resolving complex cases, especially when the clinical presentation and initial test results are inconclusive.

Bronchoscopy should not be routinely used as a means to diagnose COVID-19 infection (16). This study aims to provide knowledge for the effective and safe use of bronchoscopy in patients with suspected or confirmed COVID-19 infection. The primary objective is to ensure maximum safety for our patients, the healthcare workers who care for them, and the broader community.

We present nine cases of critically ill patients with CARDS who underwent bronchoscopy and molecular diagnostic techniques to determine the presence of microorganisms associated with the presence of COVID-19. This case series provides valuable insight into the role of bronchoscopy in managing severe COVID-19 cases and identifying superimposed infections, contributing to the growing body of knowledge on optimal care strategies for these complex patients.

### Case 1

A 51-year-old male patient with a history of obesity and high blood pressure was already admitted to the hospital with dyspnea, dry cough, fever, and headache for 11 days. The reverse transcription-polymerase chain reaction (RT-PCR) test for SARS-CoV-2 was positive. On day 2, the patient exhibited poor ventilatory mechanics and was transferred to the intensive care unit (ICU), where intubation and mechanical ventilation were initiated. A bronchoscopy with bronchoalveolar lavage (BAL) was performed on day 20. AL samples collected and processed by multiplex polymerase chain reaction (PCR) revealed multidrug-resistant *Acinetobacter baumannii* (1×107 copies), *Klebsiella pneumoniae* carbapenemase class A and D OXA (23 and 54 copies), *Candida albicans* (1×10^3^ copies), and *Chryseobacterium indologenes*. Until day 23, the patient remained on invasive mechanical ventilation (IMV).

The patient’s condition progressed unfavorably, with progressive deterioration of renal function leading to refractory septic shock and culminating in multiple organ failure. He died 47 days after his admission to the ICU.

### Case 2

A 74-year-old female patient with a history of hypertension was presented to the hospital with dyspnea, cough, fever, diarrhea, and 80% saturation. An RT-PCR test for SARS-CoV-2 performed 7 days earlier returned a positive result. On day 1, supplemental oxygen was administered through a high-flow nasal cannula (HFNC) at a rate of 50 L/min and FiO2 **of** 60%, leading to an increase in oxygen saturation to 94%. On day 15, the patient became tachypneic and unable to **tolerate** the HFNC therapy; therefore, she was transferred to the ICU, requiring intubation and connection to mechanical ventilation under protective pulmonary ventilation parameters. A bronchoscopy with BAL was performed. Samples collected during the procedure were tested using PCR techniques, and *Klebsiella pneumoniae* **carbapenemase** class A and *Acinetobacter guillouiae* were isolated. On day 30, the patient presented with poor hemodynamic ventilation parameters, requiring greater vasopressor support and 100% FiO2, as well as very high airway pressures. The patient developed anuria and refractory metabolic acidosis, which led to multisystemic organ failure. The patient died within a few hours.

### Case 3

A 68-year-old male patient without any significant medical history was admitted to the hospital with dyspnea, cough, fever, and oxygen saturation of 90% while using a mask and a reservoir at a flow rate of 15 liters per min. An RT-PCR test for SARS-CoV-2 performed 15 days earlier was obtained, showing a positive result. Supplemental oxygen was switched on and administered through an HFNC at a rate of 60 L/min, resulting in an oxygen saturation of 94%. On day 13, the patient developed symptoms of acute respiratory failure, which required oxygen at an FiO_2_ of 80%. Therefore, he was transferred to the ICU where intubation was performed. On day 14, the patient exhibited persistent respiratory acidosis in gasometric controls despite previous measures, for which extracorporeal membrane oxygenation (ECMO) therapy was initiated. In the following days, the patient exhibited improved gasometric values but was unable to handle lower levels of sedation. On day 24, a tracheostomy was performed and a chest X-ray was conducted due to fever peaks, which showed an increase in bilateral bi-basal pulmonary condensation infiltrates.

On day 28, the bronchoscopy revealed evidence of clots along with areas of erythematous and hemorrhagic dots and minimal mucous secretion. Later, a BAL sample collected and tested through PCR testing revealed *Pseudomonas aeruginosa* and *Klebsiella pneumoniae* carbapenemase. The BAL culture isolated *Burkholderia cenocepacia,* which was resistant to ceftazidime but sensitive to meropenem and minocycline.

On day 53, during extracorporeal membrane oxygenation (ECMO) therapy, the patient presented with bleeding from the femoral cannula, which was controlled after hemostatic measures were applied. An echocardiogram was performed showing a decrease in left ventricular ejection fraction (LVEF) to 43%, which was previously 60%, and an increase in pulmonary artery systolic pressure (PASP) to 48 mmHg. The patient’s clinical condition worsened and progressed to multiple organ failure, ultimately resulting in his death the following day.

### Case 4

A 51-year-old female patient with no significant medical history was admitted to the hospital with dyspnea, asthenia, cough, expectoration, fever, and oxygen saturation of 90%. The result of an RT-PCR test for SARS-CoV-2 was positive. On day 3, the patient was transferred to the ICU due to failure to tolerate HFNC therapy, which prompted intubation and mechanical ventilation. On day 6, a BAL sample was collected. The sample was tested using PCR techniques and revealed *Klebsiella pneumoniae* carbapenemase class A. The galactomannan test was positive for *Aspergillus*. On day 10, diminished alveolar opacities were observed in both lungs, and the patient was extubated on day 16 and then connected to an HFNC. After receiving a fraction of inspired oxygen (FiO2) of 60%, the patient exhibited good ventilatory mechanics. After 3 days, high broad-spectrum antibiotics were suspended, and the patient was transferred to an intermediate care unit. On day 27, the patient, exhibiting a good prognosis, was switched from the HFNC to a simple nasal cannula, maintaining oxygen saturation of 94–96%. By day 32, the patient was discharged, and pulmonary rehabilitation was recommended, along with the use of supplemental oxygen through a nasal cannula at 2 L/min to maintain a SaPO2 level of 94–94% until total weaning.

### Case 5

A 61-year-old female patient without a significant medical history was admitted to the hospital with fever, headache, arthromyalgia, anosmia, dry cough, diarrhea, and oxygen saturation of 94%. The result of an RT-PCR test for SARS-CoV-2 was positive. On day 2, the patient exhibited deterioration in lung function, with increased dyspnea and desaturation of up to 85%, prompting the initiation of HFNC therapy. The patient was transferred to the ICU, where intubation was performed, and placed on IMV. Soon after, a bronchoscopy with BAL was performed. SARS CoV-2 was isolated from the sample collected, and using PCR techniques, a viral load of 9,904 × 106 copies/μL was detected. On day 8, extubation was performed, and the patient was switched to HFNC with a FiO2 of 60% and a saturation of 96%. After a few days of improvement on the nasal cannula alone, the patient was finally discharged on day 18 with SaO2 >94% without any oxygen support and in good clinical condition.

### Case 6

A 71-year-old male patient with a history of hypertension and type 2 diabetes mellitus was admitted to the ICU of the hospital with a 9-day history of respiratory symptoms that had been previously treated at a private clinic where tocilizumab was administered 24 h before arrival. The result of an RT-PCR test for SARS-CoV-2 was positive.

On day 1, supplemental oxygen was administered through an HFNC without success, which prompted a switch to intubation and invasive mechanical ventilation. On day 3, a bronchoscopy with BAL was performed, and the sample was collected and sent to the molecular biology laboratory. Using multiplex PCR and mass spectrometry, *Trichosporon asahii*, *Klebsiella pneumoniae,* a KPC-producing strain, *Pseudomonas aeruginosa,* and *Aspergillus* were detected. These were identified by a galactomannan test, with a positive rate of 0.65. Antibiotic therapy was rotated; however, the patient developed multiple organ failure and eventually passed away.

### Case 7

A 45-year-old male patient with a history of grade II obesity was admitted to the ICU due to respiratory symptoms that had developed over 10 days. He had been treated at home with penicillin and corticosteroids. He started experiencing respiratory difficulties 24 h before being admitted and tested positive for COVID-19 via PCR swab. Support was provided with high-flow nasal cannula oxygen therapy, which eventually failed, requiring invasive mechanical ventilation. On day 3 of the ICU admission, a fibrobronchoscopy with BAL was performed. The BAL sample was sent to the molecular biology laboratory, where *Staphylococcus aureus* was detected using multiplex PCR and mass spectrometry. mecA was resistant to vancomycin, and galactomannan was *Aspergillus* positive. Targeted antibiotic therapy with linezolid and voriconazole was started. He recovered favorably, with a decrease in acute phase reactants. He was weaned from mechanical ventilation and decannulated without complications. After 25 days of hospitalization in the ICU, he was discharged.

### Case 8

A 55-year-old female patient with a history of depressive disorder tested positive for COVID-19 via PCR swab. She was admitted due to respiratory symptoms that had developed over 7 days, with the main concerns being persistent fever and worsening cough. She was transferred to the ICU, where she received support through a high-flow nasal cannula, which failed, prompting the initiation of IMV. On day 3 of the ICU admission, BAL was performed. No pathogens were detected using multiplex PCR and mass spectrometry. She died 72 h later due to refractory hypoxemia and multiple organ failure.

### Case 9

A 66-year-old female patient with no pathological history of clinical importance was brought to the emergency service in a state of cardiorespiratory arrest, for which she received advanced cardiopulmonary resuscitation for 8 min and successfully regained spontaneous circulation. She was transferred to the ICU and placed on invasive mechanical ventilation. The results of the RT-PCR test for SARS-CoV-2 were positive.

On the 13th day in the ICU, a bronchoscopy with BAL was performed. Using multiplex polymerase chain reaction, multiresistant *Acinetobacter baumannii* was detected in the BAL, which was sensitive to colistin. Therefore, 100 mg of colistin was started every 12 h.

On the 17th day in the ICU, the patient’s clinical condition worsened, and she died 48 h later.

**Figure 1:**
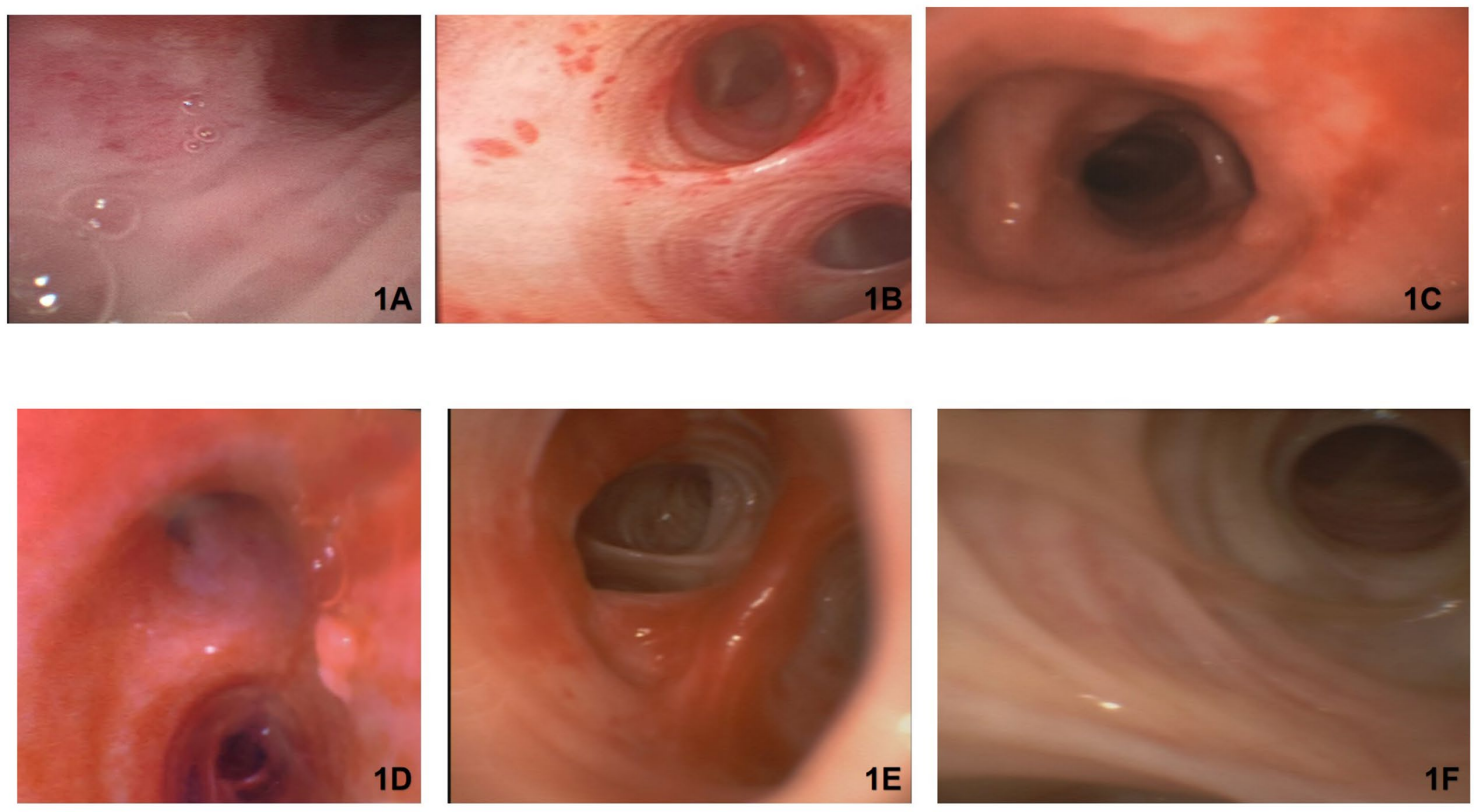
Bronchoscopic images provide valuable insights into the pathological changes that occurred in the airways of the patients with severe COVID-19. **(A)** Shows mucosal redness in the anterior wall of the subglottic region of the trachea, suggesting an infectious process. This finding is consistent with the known tropism of SARS-CoV-2 for the respiratory epithelium. **(B,D)** Show mucosal redness resulting from hemorrhagic bronchitis, characterized by small, confluent hemorrhagic areas that resemble “flea bites” on the tracheal mucosa. This finding may be a result of the increased vascular permeability and microvascular damage associated with COVID-19. **(C)** Shows edema and redness of the anterior wall of the right upper lobe bronchus, along with a dilated mucous gland orifice and mucopurulent secretions in the posterior segment. These changes suggest an ongoing inflammatory process and the presence of a secondary bacterial infection. **(E)** Shows irregularity of the mucosa in the left upper lobe, accompanied by vascular congestion and tortuosity. These findings are indicative of the extensive inflammatory response and microvascular alterations in the airways of patients with CARDS. **(F)** Shows blurred or indistinct cartilage in the left main bronchus and right lateral wall, which may be the result of pronounced mucosal edema and inflammation.

**Figure 2:**
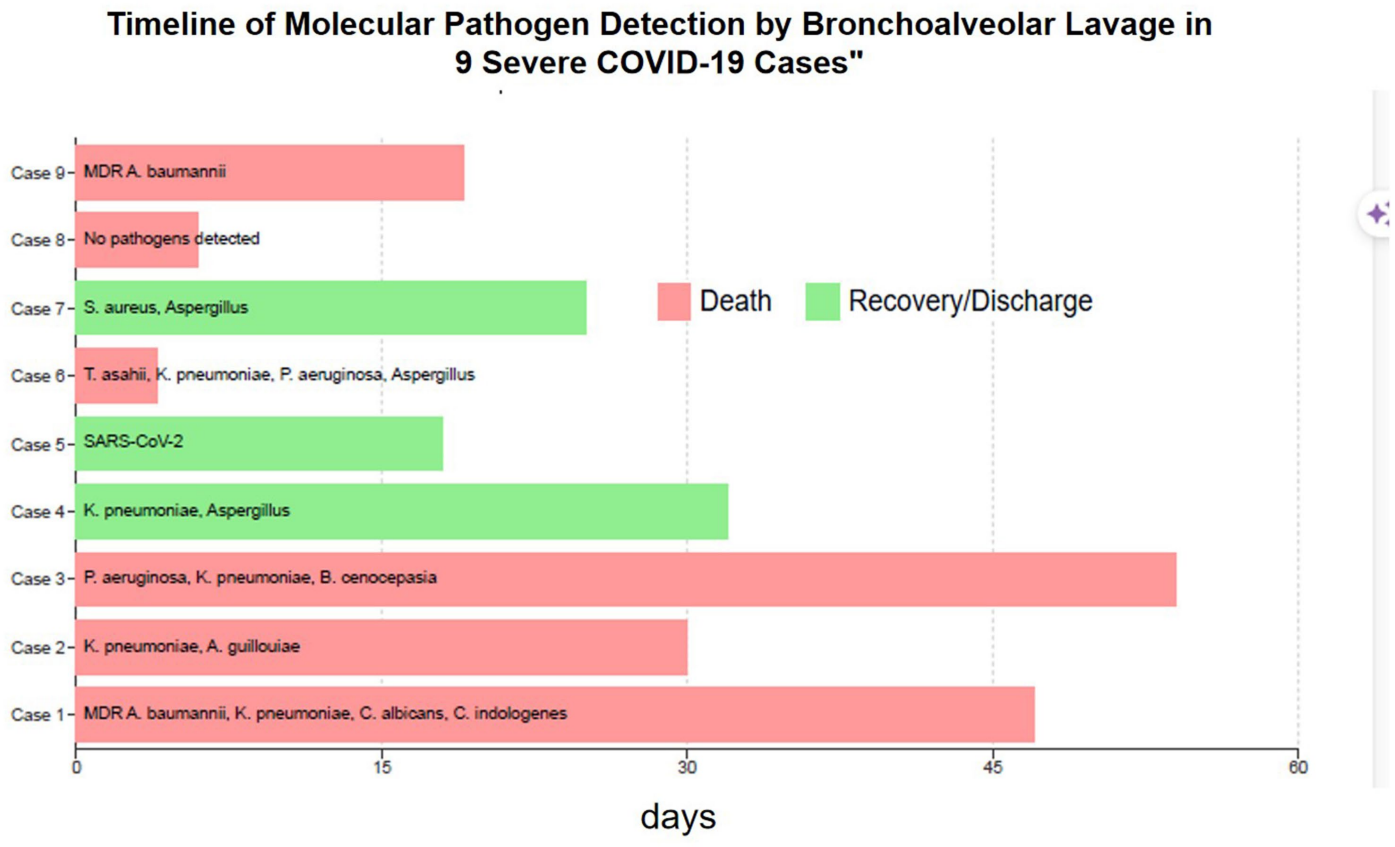
Timeline of the molecular pathogen detection by the bronchoalveolar lavage in the nine severe COVID-19 cases. This figure presents a detailed timeline of the key clinical events for the nine severe COVID-19 cases with superinfections. Each horizontal line represents an individual patient’s hospitalization timeline, starting from the day of admission (A) and covering the entire duration of their hospital stay. Color Coding: Light Red Bar: Indicates the cases that resulted in patient death. Light Green Bar: Represents the cases that resulted in patient recovery and discharge. X-axis: Labeled “Days, “representing the duration of hospital stay. Y-axis: Displays case numbers, ranging from “Case 9” at the top to “Case 1” at the bottom. Bar Contents: The text within each bar indicates the pathogens detected via bronchoalveolar lavage (BAL) for each case. Pathogen Abbreviations: A comprehensive list below the graph explains the abbreviations used for the detected pathogens: DR: Multidrug-resistant; baumannii: *Acinetobacter baumannii*; pneumoniae: *Klebsiella pneumoniae*; albicans: *Candida albicans*; indologenes: *Chryseobacterium indologenes*; guillouiae: *Acinetobacter guillouiae*; aeruginosa: *Pseudomonas aeruginosa*; cenocepacia: *Burkholderia cenocepacia*; aureus: *Staphylococcus aureus*; asahii; *Trichosporon asahii*; and RS-CoV-2: Severe acute respiratory syndrome coronavirus 2.

During fiberoptic bronchoscopy, the ventilator settings were standardized as follows: FiO2 was increased to 100%, tidal volume (TV) set to 6–8 mL/kg of ideal body weight, respiratory rate (RR) set to 20 breaths per min, level of positive end-expiratory pressure (PEEP) set to 5 cm H2O, pressure limit set to 60 cm H2O, and the maximum inspiratory flow rate was set to 50 with a ramp wave and additional leak compensation. Furthermore, 15 min before the start of the procedure, the FiO2 was set to 100% and remained at that level throughout the procedure. During fiberoptic bronchoscopy, all ventilatory parameters remained constant (Figures 1, 2 and Table 1).

**Table 1:** The baseline characteristics, laboratory tests, blood biomarkers, tomographic characteristics, and treatments associated with the isolated pathogens of each of the patients.

Clinical Characteristics	Patient 1	Patient 2	Patient 3	Patient 4	Patient 5	Patient 6	Patient 7	Patient 8	Patient 9
Age	51	74	68	51	61	71	45	55	66
Sex	Male	Female	Male	Female	Female	Male	Male	Female	Female
Past illness history	Hypertension*, Obesity	Hypertension	None	None	None	Hypertension, Diabetes type II	Obsesity type II	Depression	None
Disease history days prior hospital admission	11	7	16	5	7	9	10	7	12
Symptoms	Fever, dyspnea, dry cough, malaise, polyarthralgia.	Fever, dry cough, dyspnea, diarrhea.	Fever, dry cough, dyspnea, malaise.	Fever, asthenia, cough with expectoration	Fever, headache, arthromyalgia, anosmia, dry cough, and diarrheal stools	Fever, cough, dyspnea, malaise	Odynophagia, fever, dyspnea, malaise	Fever, odynophagia, dyspnea, malaise	Fever, malaise, dyspnea, cough, cardiorespiratory arrest during admission
RT-PCR Test for SARS-CoV-2	Positive	Positive	Positive	Positive	Positive	Positive	Positive	Positive	Positive
Oxygen saturation with HFNC device (%)	80	75	88	80	80	92	88	90	Not used*
Laboratory findings									
White cell count (per mm^3^) (normal range 4,400 to 10,300)	23,790	20,000	20,340	15,320	11,260	25.20	15.11	26.27	19,950
Total neutrophils (normal range 1780 to 5,380)	22,410	18,550	19,270	14,160	10,730	24.30	13.90	22.89	16.95
Total lymphocytes (normal range 1,180 to 3,740)	430	870	530	740	210	520	770	261	243
CRP (mg/dl) (normal range 0 to 5)	44.31	8.45	43.98	4.25	27.82	53.20	−	16	34.54
Ferritin (ng/ml) (normal range 30 to 400)	1,736	2,221	1,435	1,133	1719	>2000	>2000	964	6,828
**IL-6 (pg/ml) (normal range 0 to 6.5)	149.20	18.75	1,351	38.97	107.8	4,365	282	1,384	986.4
D-dimer (mg/l) (normal range 0 to 1.9)	0.79	2.95	4.14	2.59	0.60	6.8	1.18	9.2	4.27
Procalcitonine (ng/ml) (normal range < 0.046)	5.34	0.07	1.79	0.10	0.27	1.34	−	0.16	66.88
Radiological (CT-scan) findings:									
Ground-glass opacity	+++	+++	+++	++	+++	+++	+++	+++	+++
Condensation areas	Yes	No	Yes	Yes	Yes	Yes	Yes	No	Yes
Crazy paving pattern	No	+	No	+	+	+	No	No	+
Solitary nodule	No	No	No	No	No	No	No	No	No
Bronchoalveolar lavage findings									
Microorganisms	*Klebsiella pneumoniae* carbapenemase class A and class D OXA; Carbapenem-multiresistant *Acinetobacter baumannii* class D OXA 23 and class D OXA 51 *Candida albicans*, Cryseobacterium indologenes, Apergillus sp.	*Klebsiella pneumoniae* carbapenemase class A; *Acinetobacter guillouiae*.	Pseudomona aeruginosa; *Klebsiella pneumoniae* Carbapenemase; *Burkholderia cenocepacia*; *Candida albicans*; Aspergillus sp	GES-type carbapenemase-producing *P. aeruginosa*; Aspergillus	SARS CoV-2	Trichosporon asahii, Aspergillus; Klebsiella; pneumoniae carbapenemase; Pseudomonas; aeruginosa.	*Staphylococcus aureus* mecA; Aspergillus	SARS CoV-2	*Acinetobacter baumannii*
Prescription	Supplemental oxygen (HFNC), meropenem, moxifloxacin, tigecycline, ceftazidime / avibactam, trimethoprim / sulfamethoxazole, unfractionated heparin, norepinephrine.	Supplemental oxygen (HFNC), meropenem, voriconazole, moxifloxacin, ivermectin, nebulized amikacin, enoxaparin, prednisone, atorvastatin, colchicine, antihypertensives	Supplemental oxygen (HFNC), received tocilizumab prior to their ICU admission. Colistin, tigecycline, meropenem, voriconazole, nebulized amikacin, enoxaparin, colchicine, ivermectin, atorvastatin, antihypertensives.	Supplemental oxygen (HFNC), colistin, minocycline, ceftazidime / avibactam, fosfomycin and voriconazole, rendisivir, dexamethasone, colchicine, atorvastatin, meropenem, and moxifloxacin	Supplemental oxygen (HFNC), dexamethasone, enoxaparin, atorvastatin, piperacillin / tazobactam, and moxifloxacin.	Received tocilizumab prior to their ICU admission. Mechanical ventilation, imipenem + cilastatin, levofloxacin, dexamethasone, enoxaparin, simvastatin	Mechanical ventilation, cefepime, levofloxacin, dexamethasone, enoxaparin, simvastatin.	Received tocilizumab prior to their ICU admission Mechanical ventilation, meropenem, vancomycin, voriconazole, dexamethasone, enoxaparin, simvastatin	Mechanical ventilation, meropenem, vancomycin, voriconazole, dexamethasone, enoxaparin, simvastatin
Mechanical ventilation needed	Yes	Yes	Yes	Yes	Yes	Yes	Yes	Yes	Yes
Survival	No	No	No	Yes	Yes	No	Yes	No	No

*Hypertension, high blood pressure; **C-RP, C reactive protein; ***IL-6, Interleukinae 6; ****HFNC, High flow nasal cannula.

## Discussion

In our case series of nine critically ill patients with COVID-19-associated ARDS (CARDS), we observed that SARS-CoV-2 infection can be associated with other microorganisms, highlighting the importance of early detection of secondary infectious etiologies. In this context, bronchoscopy is emerging as a valuable alternative for the early detection of secondary infectious etiologies and appropriate microbiological rescue (17).

Our findings corroborated that coinfections and superinfections in COVID-19 patients are associated with poor outcomes, including higher mortality rates and an increased need for invasive mechanical ventilation (IMV) (18). By focusing on identifying these infections through bronchoscopy and molecular diagnostic techniques, our case series addressed a critical aspect of COVID-19 management.

In our case series, the proper identification of coinfections and superinfections required pathogen isolation through microbiological culture from respiratory or blood samples, complemented by other molecular diagnostic techniques such as antigen detection or PCR for respiratory pathogens. We identified and reported on associated pathogens in our series of nine patients with coinfections and superinfections due to SARS-CoV-2 infection.

Although our case series focused on COVID-19, it is pertinent to consider insights from other viral infections. Previous studies have shown that after influenza virus infection, there is a reduced susceptibility to superinfections These findings may have implications for understanding the immune response to COVID-19 and its impact on secondary infections. However, further research is needed to confirm this finding in the context of SARS-CoV-2 infection.

Consistent with previous studies, we detected the presence of superinfections in the lower respiratory tract in patients with COVID-19, especially infections associated with *A. baumannii* and *S. aureus.* We employed early detection methods such as reverse transcription polymerase chain reaction (RT-PCR) (19). These pathogens were also identified through conventional culture, particularly *Staphylococcus aureus*, *Pseudomonas aeruginosa*, and *Klebsiella* spp. (20).

Notably, we observed carbapenem-resistant *Acinetobacter baumannii* (CRAB) in three of our patients. This finding aligns with several studies demonstrating how CRAB represents a common threat in the hospital setting, persisting on dry surfaces for extended periods and resisting the majority of disinfectants. This translates to one of the leading causes of healthcare-associated infections, thus posing a risk for higher mortality rates in ICU patients.

A key strength of our case series is the use of molecular diagnostic techniques **(****21****)** for early specimen detection. This is vital because decisions regarding antibiotic treatments in severely ill patients often must be made without microbiological results.

All patients in our series received dexamethasone as an immunomodulatory therapy to reduce the inflammatory cascade. However, according to previous studies, inflammatory biomarkers such as C-reactive protein and procalcitonin may not be useful in identifying secondary infections in severely ill patients with SARS-CoV-2, similar to patients without infection. This presents a challenge at ICU admission since biomarker levels guide treatment initiation and continuation.

Our case series revealed co-infections in two of the patients, with *Pseudomonas and Aspergillus* being the most frequent. Regarding superinfections, we observed ESBL-and KPC-producing microorganisms, especially *Klebsiella pneumoniae* and *Pseudomonas aeruginosa* (22).

Although not observed in our case series, other microorganisms, particularly tuberculosis and HIV, have been reported in COVID-19 patients in other studies (23).

Despite the percentage of the presence of co-infections in our case series, the use of empirical antibiotics was very high (24), highlighting the potential overuse of antibiotics in COVID-19 (25). In some studies, leukocytosis increased absolute neutrophil count, and procalcitonin levels >0.5 ng/mL have been associated with a high probability of bacterial infection. However, these parameters lack sufficient sensitivity, specificity, and positive predictive value to accurately diagnose bacterial coinfections as independent tests, which is associated with a higher probability of treatment failure, especially in patients receiving immunomodulators, such as tocilizumab and corticosteroids (26).

It is worth noting that three of our cases (3, 6, and 8) received tocilizumab before their ICU admission. This biologic agent, which is known for increasing infection risk, was administered when these patients had high IL-6 levels, with the aim of managing the hyperinflammation associated with severe COVID-19. The use of tocilizumab before ICU admission highlights the delicate balance between early intervention in the inflammatory cascade and the risk of secondary infections. Our case series suggests careful monitoring for superinfections in patients treated with biologics, especially those requiring ICU care. The three patients in our cohort who received tocilizumab also had the highest levels of IL-6, a finding reflected in the literature. Recent research, including the study by Horby et al. (27), has shown that tocilizumab reduces mortality in hospitalized COVID-19 patients with hypoxia and systemic inflammation. However, the COVACTA trial (28) found no improvement in clinical outcomes or mortality with early tocilizumab use in severe COVID-19 pneumonia. Regarding superinfections, Kimmig et al. (29) reported no significant increase in secondary infections in tocilizumab-treated COVID-19 patients; however, vigilance is advised. Our small case series contributes to the discussion on tocilizumab use in severe COVID-19, emphasizing careful patient selection and monitoring for complications such as superinfections. Although causality cannot be established, these findings highlight challenges in managing severe COVID-19 and raise concerns about the use of biologics such as tocilizumab.

Our findings put into perspective the fact that the clinical course of ARDS in COVID-19 is usually marked by multiple superimposed infections, leading to high mortality rates. This observation emphasizes the importance of preventive measures, particularly vaccination, in containing the pandemic and reducing the incidence of COVID-19-induced ARDS. Seminal studies have shown that vaccination against COVID-19 is safe and effective in preventing severe disease (30). Moreover, population-based analyses have shown much better clinical outcomes in vaccinated patients compared to their unvaccinated counterparts. Such findings emphasize the critical role played by vaccination in the reduction of severe consequences of COVID-19, such as ARDS and associated superinfections. Our case series contributes significant insight as it was conducted at a time when vaccines were not widely available, suggesting that more widespread vaccination could potentially have averted such severe cases (31).

Our case series, conducted during the second wave of COVID-19 in Ecuador, acknowledges the unique conditions and challenges faced in resource-limited settings. The constraints of such environments necessitate innovative approaches and adaptation of standard practices to ensure effective patient management, particularly in severe COVID-19 cases where resources are limited (32).

The lessons learned from this case series are pivotal in the current pandemic context and have significant implications for future health crises. Understanding the dynamics and management strategies in resource-constrained settings, as observed in our patients from Ecuador, provides valuable insights into global health, emphasizing the need for adaptable and scalable solutions during pandemics (33).

Limitations and Future Research Directions: A notable limitation of this case series is the lack of a standard and consensus definition for the criteria determining the start and end of a COVID-19 epidemic wave (34). This has led to discrepancies in the established dates compared to other studies. In addition, accurately identifying the end of one wave and the beginning of the next is complex as virus transmission persists at baseline levels (35). This study relied on confirmed case data, which can be influenced by external factors, such as variations in diagnostic capacity over time. Furthermore, the small number of cases in our series limits the generalizability of our findings. In addition, further research is needed to evaluate the long-term outcomes of patients with identified coinfections and superinfections.

In conclusion, this case series highlights the utility of bronchoscopy and molecular diagnostic techniques in detailing bronchial mucosal characteristics, underscoring their critical role in diagnosing and managing CARDS by identifying airway involvement and secondary infections. These techniques are particularly valuable when certain clinical and laboratory parameters are present in critically ill patients. Our findings suggest that BAL and the early use of molecular diagnostic techniques play an important role in the management of severe COVID-19 cases, particularly in resource-limited settings. Future studies should focus on optimizing these techniques and developing standardized protocols for their use in pandemic situations and should include larger patient populations to validate and expand upon our observations.

## Data Availability

The original contributions presented in the study are included in the article/Supplementary material, further inquiries can be directed to the corresponding author/s.
